# Author Correction: MiDAS 4: A global catalogue of full-length 16S rRNA gene sequences and taxonomy for studies of bacterial communities in wastewater treatment plants

**DOI:** 10.1038/s41467-022-31423-z

**Published:** 2022-07-11

**Authors:** Morten Kam Dahl Dueholm, Marta Nierychlo, Kasper Skytte Andersen, Vibeke Rudkjøbing, Simon Knutsson, MiDAS Global Consortium, Sonia Arriaga, Rune Bakke, Nico Boon, Faizal Bux, Magnus Christensson, Adeline Seak May Chua, Thomas P. Curtis, Eddie Cytryn, Leonardo Erijman, Claudia Etchebehere, Despo Fatta-Kassinos, Dominic Frigon, Maria Carolina Garcia-Chaves, April Z. Gu, Harald Horn, David Jenkins, Norbert Kreuzinger, Sheena Kumari, Ana Lanham, Yingyu Law, TorOve Leiknes, Eberhard Morgenroth, Adam Muszyński, Steve Petrovski, Maite Pijuan, Suraj Babu Pillai, Maria A. M. Reis, Qi Rong, Simona Rossetti, Robert Seviour, Nick Tooker, Pirjo Vainio, Mark van Loosdrecht, R. Vikraman, Jiří Wanner, David Weissbrodt, Xianghua Wen, Tong Zhang, Per H. Nielsen, Mads Albertsen, Per Halkjær Nielsen

**Affiliations:** 1 Center for Microbial Communities, Department of Chemistry and Bioscience, Aalborg University, Aalborg, Denmark; 2 Environmental Science Department, The Institute for Scientific and Technological Research of San Luis Potosi (IPICYT), San Luis Potosí, Mexico; 3 Department of Process, Energy and Environmental Technology, University College of Southeast Norway, Porsgrunn, Norway; 4 Center for Microbial Ecology and Technology, Ghent University, Ghent, Belgium; 5 Institute for Water and Wastewater Technology, Durban University of Technology, Durban, South Africa; 6 Veolia Water Technologies AB, AnoxKaldnes, Lund, Sweden; 7 Department Of Chemical Engineering, Faculty of Engineering, University of Malaya, Kuala Lumpur, Malaysia; 8 Environmental Engineering, Newcastle University, Newcastle, England; 9 The Cytryn Lab, Microbial Agroecology, Volcani Center, Agricultural Research Organization, Rishon Lezion, Israel; 10 INGEBI-CONICET, University of Buenos Aires, Buenos Aires, Argentina; 11 Department of Biochemistry and Microbial Genetics, Biological Research Institute “Clemente Estable”, Montevideo, Uruguay; 12 NIREAS-International Water Research Center, University of Cyprus, Nicosia, Cyprus; 13 Environmental Engineering, McGill University, Montreal, QC Canada; 14 School of Microbiology, Universidad de Antioquia, Medellín, Colombia; 15 School of Civil and Environmental Engineering, Cornell University, Ithaca, NY USA; 16 Water Chemistry and Water Technology and DVGW Research Laboratories, Karlsruhe Institute of Technology (KIT), Karlsruhe, Germany; 17 David Jenkins & Associates, Inc, Kensington, CA USA; 18 Institute for Water Quality and Resource Management, TU Wien, Vienna, Austria; 19 Water Innovation and Research Centre, University of Bath, Bath, England; 20 Singapore Centre of Environmental Life Sciences Engineering (SCELSE) Nanyang Technological University, Singapore, Singapore; 21 Water Desalination and Reuse Center, King Abdullah University of Science and Technology (KAUST), Thuwal, Saudi Arabia; 22 Process Engineering in Urban Water Management, ETH Zürich, Zürich, Switzerland; 23 Department of Biology, Warsaw University of Technology, Warsaw, Poland; 24 Environmental Microbial Genetics Lab, La Trobe University, Melbourne, VIC Australia; 25 Technologies and Evaluation Area, Catalan Institute for Water Research, ICRA, Girona, Spain; 26 VA Tech Wabag Ltd, Chennai, India; 27 Biochemical Engineering Group, Universidade Nova de Lisboa, Lisboa, Portugal; 28 State Key Laboratory of Environmental Aquatic Chemistry, Research Center for Eco-Environmental Sciences, Chinese Academy of Sciences, Beijing, China; 29 Water Research Institute IRSA - National Research Council (CNR), Rome, Italy; 30 La Trobe University, Melbourne, VIC Australia; 31 Department of Civil and Environmental Engineering, University of Massachusetts Amherst, Amherst, MA USA; 32 Kemira Oyj, Espoo R&D Center, Espo, Finland; 33 Environmental Biotechnology, TU Delft, Delft, The Netherlands; 34 VA Tech Wabag, Philippines Inc., Makati City, Philippines; 35 Department of Water Technology and Environmental Engineering, University of Chemistry and Technology, Prague, Czech Republic; 36 Environmental Life Science Engineering, TU Delft, Delft, The Netherlands; 37 School of Environment, Tsinghua University, Beijing, China; 38 Environmental Biotechnology Lab, Department of Civil Engineering, The University of Hong Kong, Hong Kong, Hong Kong

**Keywords:** Microbial ecology, Microbial communities, Environmental biotechnology, Water microbiology

Correction to: *Nature Communications* 10.1038/s41467-022-29438-7, published online 07 April 2022.

The original version of this Article included the following errors in reference citations:

It incorrectly cited ‘Thompson, L. R. et al. A communal catalogue reveals Earth’s multiscale microbial diversity. *Nature* **551**, 457–463 (2017)’ and ‘Peterson, J. et al. The NIH Human Microbiome Project. *Genome Res*. **19**, 2317–2323 (2009)’ as Refs 18 and 19 in the Introduction, at ‘In the MiDAS project, we have thoroughly evaluated different wet-lab protocols, e.g., DNA extraction methods, and choice of amplicon primers and amplicon library preparation^17-19^.’ The two references have been removed from the correct version.

It incorrectly cited ‘Zheng, M. et al. Active ammonia-oxidizing bacteria and archaea in wastewater treatment systems. *J. Environ. Sci*. **102**, 273–282 (2021)’ and ‘Thompson, L. R. et al. A communal catalogue reveals Earth’s multiscale microbial diversity. *Nature* **551**, 457–463 (2017)’ as Refs 42 and 18 in the last paragraph of Results and Discussion, at ‘In the Danish WWTPs, we have successfully done this for groups in the Acidobacteriota^42^ based on the MiDAS 3 database^18^.’ The correct version replaces the first reference with ‘Kristensen, J. M., Singleton, C., Clegg, L.-A., Petriglieri, F. & Nielsen, P. H. High diversity and functional potential of undescribed “Acidobacteriota” in Danish wastewater treatment plants. *Front. Microbiol*. **12**, 906 (2021)’ and the second reference with Ref. 16.

It incorrectly cited ‘Rosselló-Mora, R. A., Wagner, M., Amann, R. & Schleifer, K. H. The abundance of *Zoogloea ramigera* in sewage treatment plants. *Appl. Environ. Microbiol*. **61**, 702–707 (1995)’ as Ref. 43 in the last paragraph of Results and Discussion, at ‘Our recent retrieval of more than 1000 high-quality MAGs from Danish WWTPs with advanced process design is also an important step to link identity to function^43^.’ The correct version replaces this reference with ‘Singleton, C. M. et al. Connecting structure to function with the recovery of over 1000 high-quality metagenome-assembled genomes from activated sludge using long-read sequencing. *Nature Communications* **12**, 2009 (2021)’.

It incorrectly cited ‘Nierychlo, M. et al. *Candidatus* Amarolinea and *Candidatus* Microthrix are mainly responsible for filamentous bulking in Danish municipal wastewater treatment plants. *Front. Microbiol*. 11, 1214 (2020)’, ‘Klindworth, A. et al. Evaluation of general 16S ribosomal RNA gene PCR primers for classical and next-generation sequencing-based diversity studies. *Nucleic Acids Res*. **41**, 1–11 (2013)’ and ‘Lane, D. J. 16S/23S rRNA sequencing In *Nucleic Acid Techniques in Bacterial Systematics* (eds Stackebrandt, E. & Goodfellow, M.) (Wiley, 1991)’ as Refs 44–46 in the last paragraph of Results and Discussion, at ‘These MAGs may also form the basis for further studies to link identity and function, e.g., by applying metatranscriptomics^44^ and other in situ techniques such as FISH combined with Raman^45,46^.’ The correct version replaces these references with ‘Herold, M. et al. Integration of time-series meta-omics data reveals how microbial ecosystems respond to disturbance. *Nature Communications* **11**, 5281 (2020)’, ‘Fernando, E. Y. et al. Resolving the individual contribution of key microbial populations to enhanced biological phosphorus removal with Raman–FISH. *ISME J*. **13**, 1933–1946 (2019)’ and ‘Petriglieri, F. et al. Quantification of Biologically and Chemically Bound Phosphorus in Activated Sludge from Full-Scale Plants with Biological P-Removal. *Environ. Sci. Technol.* **56**, 5132–5140 (2022)’.

The errors have been corrected in the PDF and HTML versions of the Article.

