# Supplementary material for: MiDAS 4: A global catalogue of full-length 16S rRNA gene sequences and taxonomy for studies of bacterial communities in wastewater treatment plants
Source: Nat Commun. 2022 Apr 7;13:1908. doi: 10.1038/s41467-022-29438-7 (PMC8989995; doi:10.1038/s41467-022-29438-7)
Supplement: Supplementary file 1 — Supplementary information [file 41467_2022_29438_MOESM1_ESM.pdf]

Supplementary Information for:

MiDAS 4: A global catalogue of full-length 16S rRNA gene sequences and taxonomy for studies of bacterial communities in wastewater treatment plants

Authors: Morten Kam Dahl Dueholm[1,*], Marta Nierychlo[1], Kasper Skytte Andersen[1], Vibeke Rudkjøbing[1], Simon Knutsson[1], MiDAS Global Consortium[**], Mads Albertsen[1] & Per Halkjær Nielsen[1,*]

Affiliations:

[1]Center for Microbial Communities, Department of Chemistry and Bioscience, Aalborg University, Aalborg, Denmark. [**]A list of authors and their affiliations appears at the end of the main paper.

[*]Correspondence to: Per Halkjær Nielsen, Center for Microbial Communities, Department of Chemistry and Bioscience, Aalborg University, Fredrik Bajers Vej 7H, 9220 Aalborg, Denmark; Phone: +45 9940 8503; Fax: Not available; E-mail: phn@bio.aau.dk or Morten Kam Dahl Dueholm, Center for Microbial Communities, Department of Chemistry and Bioscience, Aalborg University, Fredrik Bajers Vej 7H, 9220 Aalborg, Denmark; Phone: (+45) 9940 8508; Fax: Not available; E-mail: md@bio.aau.dk

Table of content:

Page 2-4: Supplementary Note 1-4
Page 5-18: Supplementary Fig. 1-16
Page 19: Supplementary Table 1
Page 20: Supplementary References

Supplementary Notes:

Supplementary Note 1: Detailed analysis of alpha diversity

Alpha diversity analyses revealed that the richness and diversity of WWTPs were mainly determined by the process type, industrial load, and continent, whereas the temperature range and climate zone were less discriminatory (Supplementary Fig. 3). Both the richness and the diversity increased with the complexity of the treatment process as also found in other studies, reflecting the increased number of niches [1]. In contrast, it decreased with high industrial loads, reflecting that industrial wastewater often is less complex and therefore promotes growth of fewer species [2]. The highest richness and diversity were observed in Oceania and Africa, and the lowest in South America. These differences may partially be linked to differences in process type and industrial load, as the sampled WWTPs in Oceania and Africa were mainly advanced plants (C,N,DN or C,N,DN,P), receiving only little industrial wastewater. In contrast, the South American plants were simple carbon-removal plants that treated >50% industrial wastewater on average.

Supplementary Note 2: Distance decay relationship

The similarity between microbial communities is generally considered to decrease with geographic distance for most environments including WWTPs [3–6]. Therefore, we performed distance decay relationship (DDR) analyses to determine how it affects the activated sludge microbiota on a global scale (Supplementary Fig. 4). We observed similar DDR for both ASVs and OTUs (clustered at 97% identity) based on Mantel R values. In addition, we observed that distance decay was only effective within shorter geographical distances (<2,500 km), and almost absent at global scales (>2,500 km).

Although ASVs and OTUs displayed similar DDR, we observed that the Bray-Curtis and Soerensen similarities were considerably lower for ASVs than for OTUs. A complete lack of similarity was observed between many samples at the ASV resolution (similarity ≈ 0) but not for OTUs. This lack of similarity was especially pronounced for the abundance-weighted Bray-Curtis similarity, indicating that the abundant process-critical bacteria were especially geographically restricted at the ASV level (Supplementary Fig. 4).

Because of the low similarity between microbial communities at the ASV level and because OTUs are hard to compare across studies, we also investigated the effect of geographic distance on the taxonomic diversity at the genus level, where we expect that many important traits are conserved (Supplementary Fig. 4). This analysis was only possible because of the high classification rate achieved with MiDAS4. The genus level diversity was less affected by DDR compared to ASV and OTU diversity, according to lower Mantel R values. Furthermore, the genus level similarity between the global samples was also markedly higher than the ASV and OTU level diversity.

Supplementary Note 3: Detailed analysis of genus-level beta diversity

To gain a deeper understanding of the factors that shape the activated sludge microbiota, we examined the Bray-Curtis (weighted) and Soerensen (unweighted) beta diversity at the genus-level using principal coordinate (PCoA) and permutational multivariate analysis of variance (PERMANOVA) analyses (Fig. 5). The PCoA analyses revealed clear separation of the microbial communities in respect to all parameters investigated (Fig. 5). In addition, we noted clear associations between many of the process-specific and environmental factors investigated, e.g., between high industrial load and simpler process types, and not unexpectedly, between temperature range and climate zones. As a result of these associations, it is hard to accurately assign the contribution of variance by individual factors to the overall beta diversity. However, by examining how much of the total variance could be explained by each parameter isolated, it was possible to rank the strength of each parameter. We found that the overall microbial community was most strongly affected by continent and temperature in the WWTPs. However, process type, industrial load, and the climate zone also had significant impacts. The percentage of total variation explained by each individual parameter was generally low, indicating that the global WWTPs microbiota represents a continuous distribution rather than distinct states, as has also been observed for the human gut microbiota [7].

Supplementary Note 4: Redundancy analysis for industrial load, continents, and climate zones

In order to identify which genera were most affected by process and environmental factors, we performed redundancy analyses (RDA) with the relative abundance of each genus as dependent variables and the process and environmental factors as explanatory variables. The RDAs were constrained by each factor separately, and both V1-V3 (Supplementary Fig. 5) and V4 (Supplementary Fig. 6) amplicon data were analyzed to ensure that important taxa were not missed due to primer bias. RDA scores for all genera and analyses can be found in Supplementary Data 3.

When industrial WWTPs (high industrial load) were compared to municipal plants (medium load or below), a reduced abundance of genera associated with advanced process types was observed in plants with high industrial loads (high or all), and an increased abundance of *Defluviicoccus*, *Hyphomicrobium*, *Rhodoplanes*, *Planctomicrobium*, *Ca.* Alysiosphaera, *Azospirillum*, *Azovibrio*, and *Pedomicrobium* were observed. *Defluviicoccus* was also enriched in carbon removal only plants, which is the main process type for the treatment of industrial wastewater. *Hyphomicrobium* frequently occurs in WWTPs containing or supplemented with methanol due to its ability to grow on one-carbon compounds [8].

Comparison of continents revealed clear similarities between South America and Asia, and to a lesser degree between Europe and North America. Genera with increased relative abundance in South America and Asia included *Defluviicoccus*, *Ca.* Competibacter, *Thauera*, *Rhodoplanes*, *Phaeodactylibacter,* and *Chitinivorax*, whereas WWTPs in Europe and North America displayed an increased abundance of *Rhodoferax*, *Flavobacterium*, *Ca.* Microthrix, *Tetrasphaera,* and *Acidovorax*.

Climate zones showed a clear separation between tropical, dry, and continental climates, whereas the temperate climate had similarities with all the others. Tropical and dry climates were characterized by an increase in the abundance of *Ca.* Competibacter, *Defluviicoccus, Thauera*, *Rhodoplanes*, *Ottowia*, UTCFX1, and the *de novo* taxa midas_g_70, midas_g_9648, and midas_g_399. *Rhodoplanes* was the only genus that was more abundant in the tropical climate. Continental climates were characterized by an increased abundance of *Flavobacterium*, *Rhodoferax*, *Rhodobacter, Ferruginibacter, Acidovorax*, *Tetrasphaera*, and the filamentous *Leptothrix* and *Ca*. Microthrix.

Supplementary Figures:

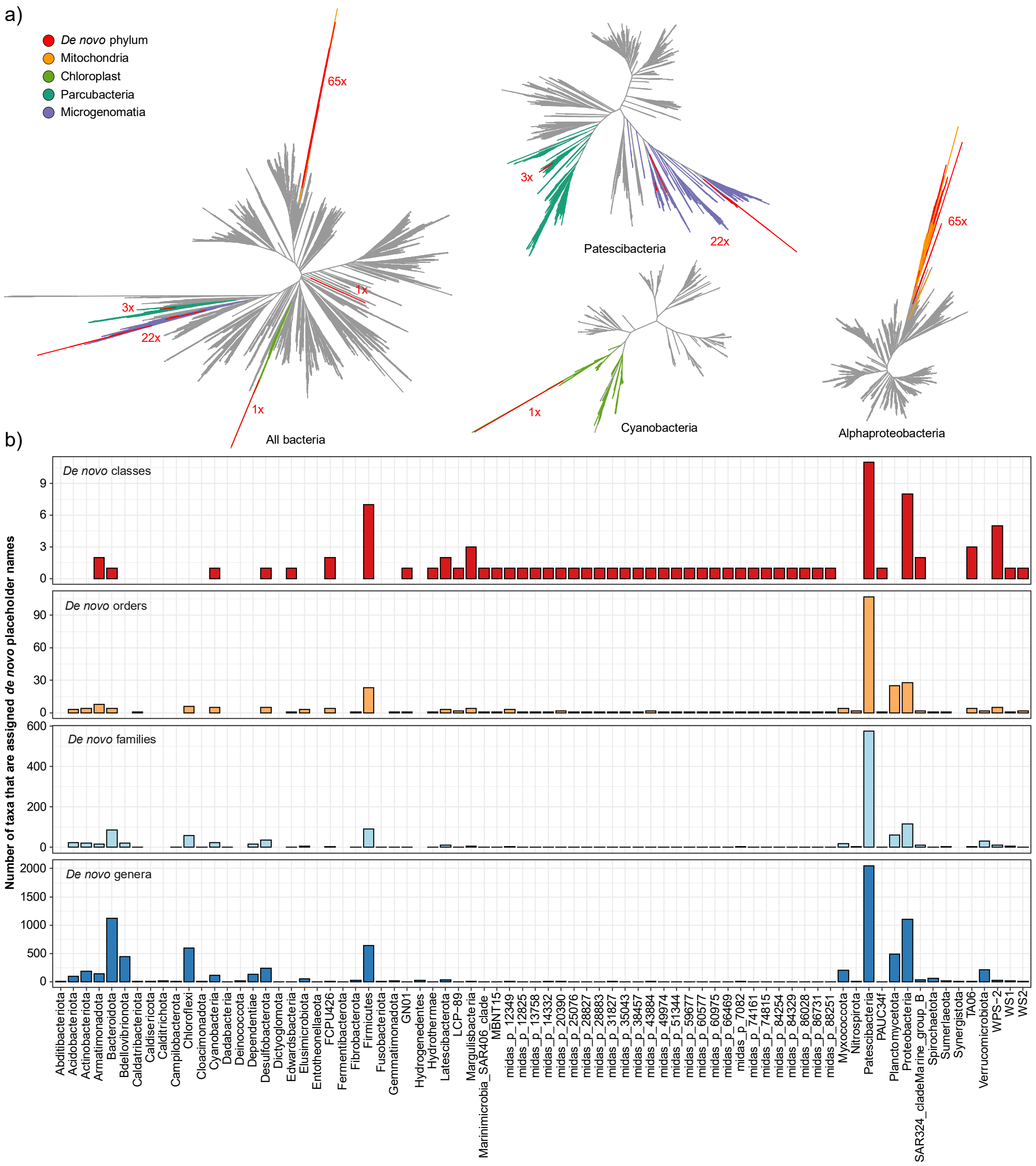

Supplementary Fig. 1: Phylogenetic and taxonomic location of *de novo* taxa. a) Phylogenetic trees showing the proposed branching point for FL-ASVs associated with *de novo* phyla. The numbers indicate how many unique FL-ASVs assigned to *de novo* phyla are found at the given location. b) The number of taxa that obtained placeholder names from the MiDAS 4 *de novo* taxonomy in different phyla.

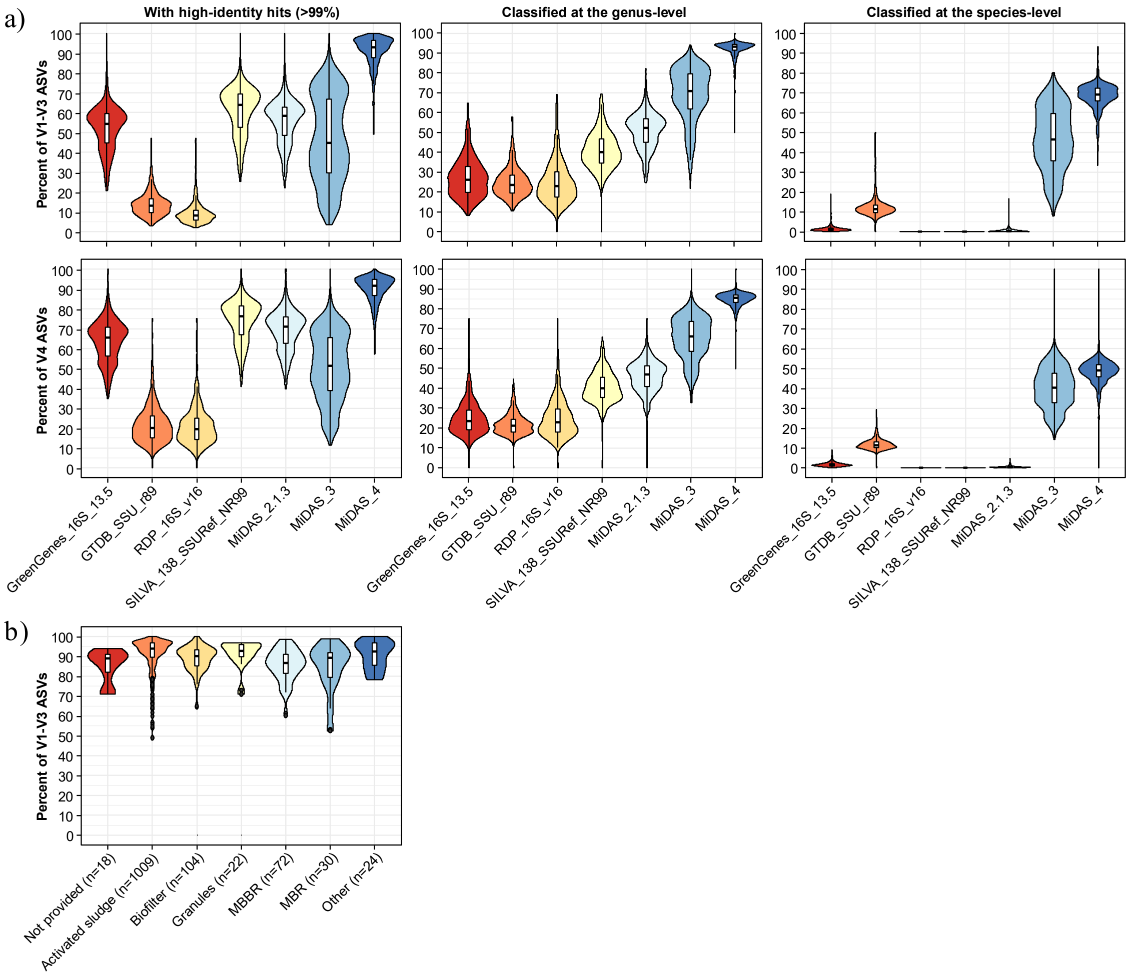

Supplementary Fig. 2: Database evaluation based on amplicon data from this study. a) V1-V3 and V4 ASVs were obtained from 1279 and 1278 samples, respectively, and filtered based on their relative abundance (≥0.01%) before the analyses. The percentage of the microbial community represented by the remaining ASVs after the filtering was 92.69% ± 3.22% and 96.4% ± 2.27% across samples for V1-V3 and V4 amplicon data, respectively. High-identity (≥99%) hits were determined by the stringent mapping of ASVs to each reference database. Classification of ASVs was done using the SINTAX classifier. The violin and box plot represent the distribution of percent of ASVs with high-identity hits or genus/species-level classifications for each database across n =1279 for V1-V3 and n = 1278 for V4 biological independent samples. Box plots indicate median (middle line), 25th, 75th percentile (box), and the min and max values after removing outliers based on 1.5x interquartile range (whiskers). Outliers have been removed from the box plots to ease visualization. Colors are used distinguish the different databases. b) Percent of V1-V3 ASVs filtered as above with high-identify (≥99%) hits across different plant types. The violin and box plots represent the distribution of percent of ASVs with high-identity hits in the MiDAS 4 database across plant types with the number of biological independent samples (n) indicated on the x-axis. The box plots are defined as above. Outliers are shown as black dots. Colors are used distinguish the different plant types. MBBR: moving bed bioreactors; MBR: membrane bioreactors.

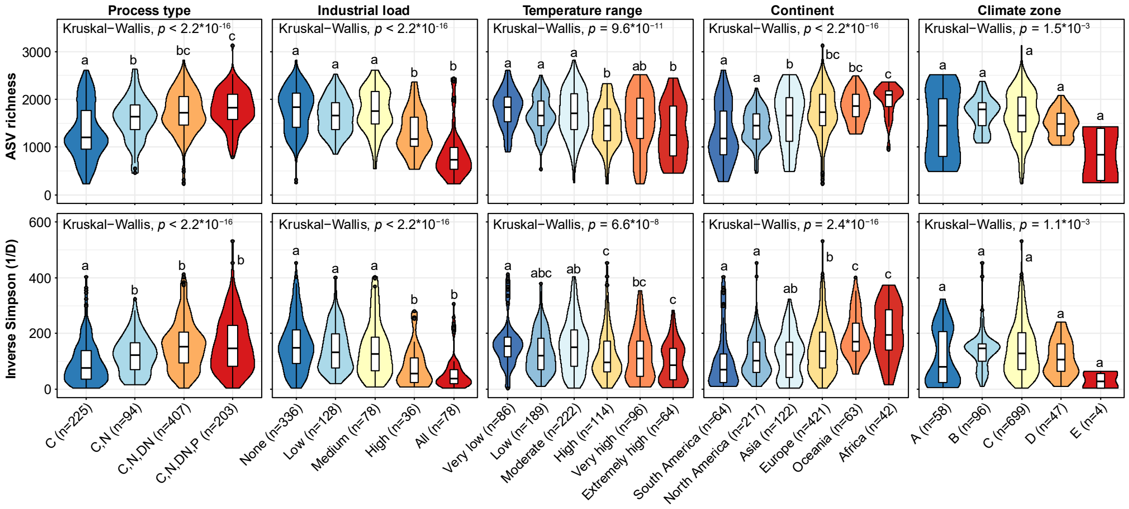

Supplementary Fig. 3: Effect of process parameters and geography on alpha diversity. The non-parametric Kruskal-Wallis test was used to determine the statistical support for differences between the means of the groupings in the V1-V3 amplicon data set. The exact value could not be determined for $p < 2.2*10^{-16}$. A post-hoc Dunn's test (Bonferroni correction, α=0.01) was used for pairwise comparison of individual groups and the results are shown with compact letter display (groups that do not share letters are significantly different). The violin and box plot represent the distribution of ASV richness or inverse Simpson (1/D) across the number of biological independent samples (n) indicated on the x-axis. The box plots indicate median (middle line), 25th, 75th percentile (box), and the min and max values after removing outliers based on 1.5x interquartile range (whiskers). Outliers are shown as black dots. Process types: C = carbon removal; C,N = carbon removal with nitrification; C,N,DN = carbon removal with nitrification and denitrification; C,N,DN,P = carbon removal with nitrogen removal and enhanced biological phosphorus removal (EBPR). Temperature range: very low = <10°C, low = 10-15°C, moderate = 15-20°C, high = 20-25°C, very high = 25-30°C, extremely high = >30°C. Industrial load: none = 0%, very low = 0-10%, low = 10-30%, medium= 30-50%, high 50-100%, all = 100%. Köppen climate classification groups: A = Tropical/megathermal climates, B = Dry (desert and semi-arid) climates, C = Temperate/mesothermal climates, D = Continental/microthermal climates, E = Polar climates. Colors match those used in Fig. 5.

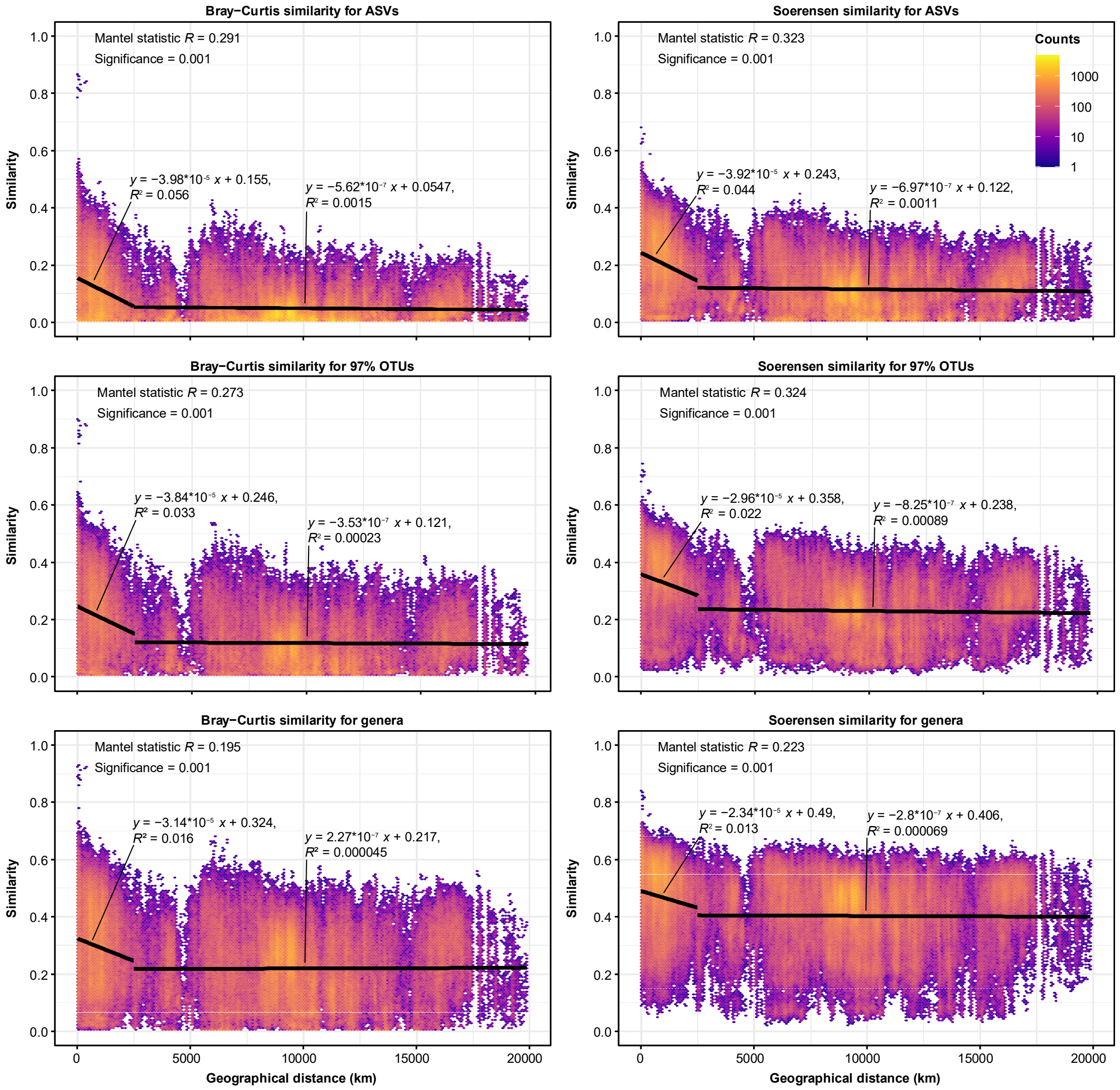

Supplementary Fig. 4: Distance decay rates based on V1-V3 amplicon data. DDR was calculated based on Bray-Curtis (weighted) and Soerensen (unweighted) distances for ASVs, OTUs clustered at 97% sequence identity, and for genus-level classifications of ASVs. Mantel statistics for the DDR is provided for each plot. Each plot also contains the equations for two linear regressions. The first represents distances below 2,500 km and the second distances above 2,500 km.

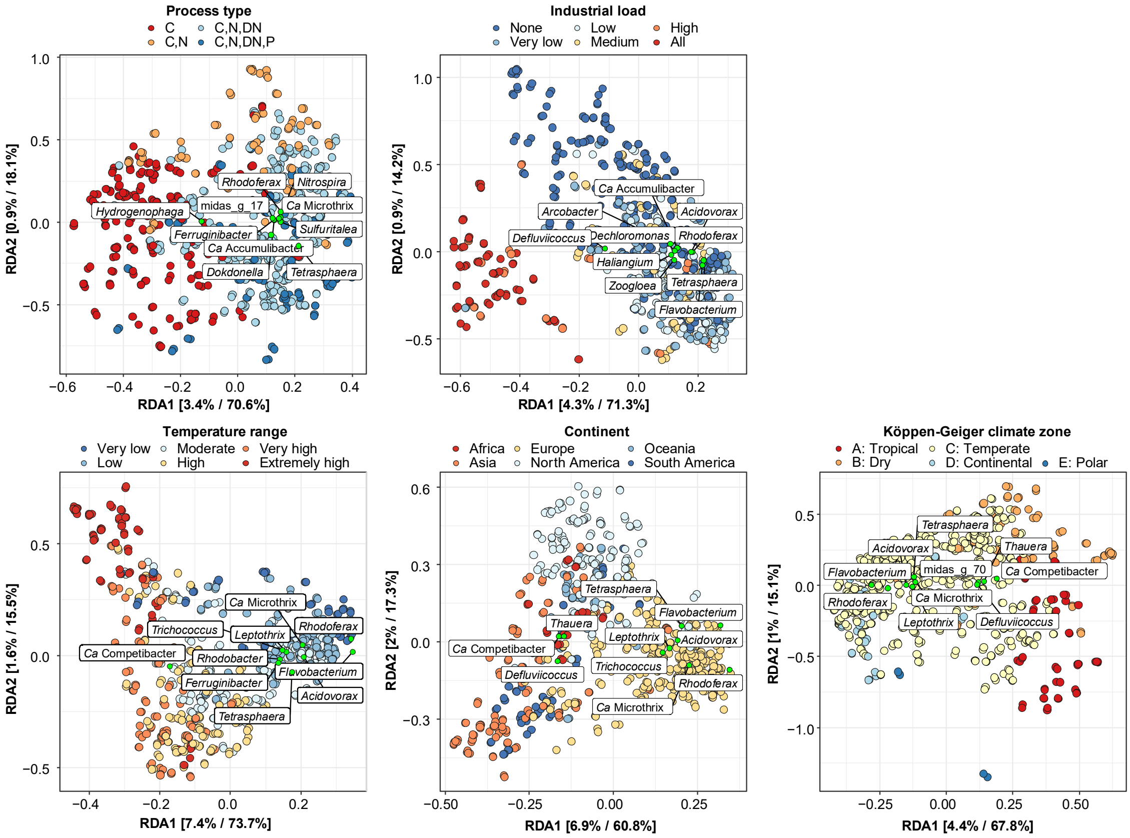

Supplementary Fig. 5: Redundancy analyses (RDA) based on V1-V3 amplicon data classified at the genus-level constrained to each of the environmental or geographical parameters separately. The data has been transformed initially by applying the Hellinger transformation. The relative contribution (eigenvalue) of each axis to the total inertia in the data, as well as to the constrained space only, respectively, are indicated in percent at the axis titles. Samples are colored based on metadata. The coordinates (green points) and names of the 10 most influential genera in relation to RDA1 is shown for each parameter. Process types: C = carbon removal; C,N = carbon removal with nitrification; C,N,DN = carbon removal with nitrification and denitrification; C,N,DN,P = carbon removal with nitrogen removal and enhanced biological phosphorus removal (EBPR). Temperature range: Very low = <10°C, low = 10-15°C, moderate = 15-20°C, high = 20-25°C, very high = 25-30°C, extremely high = >30°C. Industrial load: None = 0%, very low = 0-10%, low = 10-30%, medium= 30-50%, high 50-100%, all = 100%. A comprehensive list with RDA scores for all genera can be found in Supplementary Data 3.

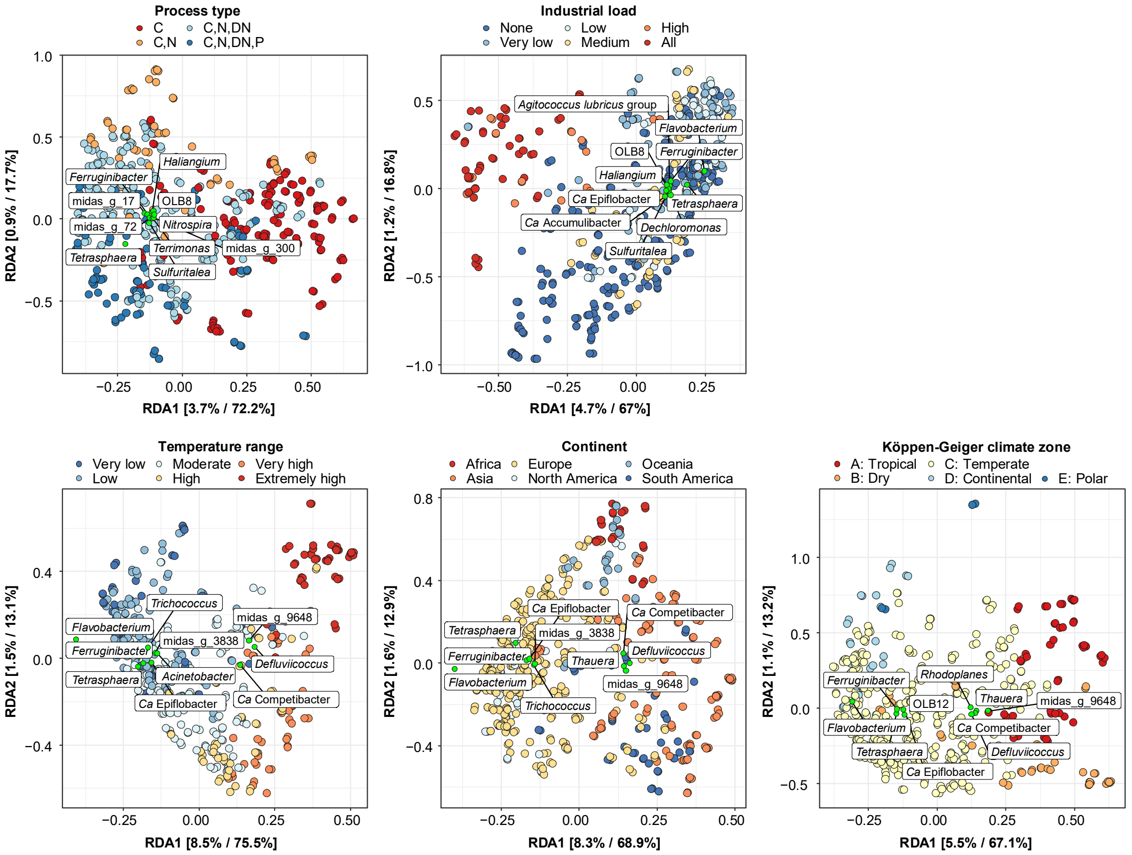

Supplementary Fig. 6: Redundancy analyses (RDA) based on V4 amplicon data classified at the genus-level constrained to each of the environmental or geographical parameters separately. The data has been transformed initially by applying the Hellinger transformation. The relative contribution (eigenvalue) of each axis to the total inertia in the data as well as to the constrained space only, respectively, are indicated in percent at the axis titles. Samples are colored based on metadata. The coordinates (green points) and names of the 10 most influential genera in relation to RDA1 is shown for each parameter. Process types: C = carbon removal; C,N = carbon removal with nitrification; C,N,DN = carbon removal with nitrification and denitrification; C,N,DN,P = carbon removal with nitrogen removal and enhanced biological phosphorus removal (EBPR). Temperature range: Very low = <10°C, low = 10-15°C, moderate = 15-20°C, high = 20-25°C, very high = 25-30°C, extremely high = >30°C. Industrial load: None = 0%, very low = 0-10%, low = 10-30%, medium= 30-50%, high 50-100%, all = 100%. A comprehensive list with RDA scores for all genera can be found in Supplementary Data 3.

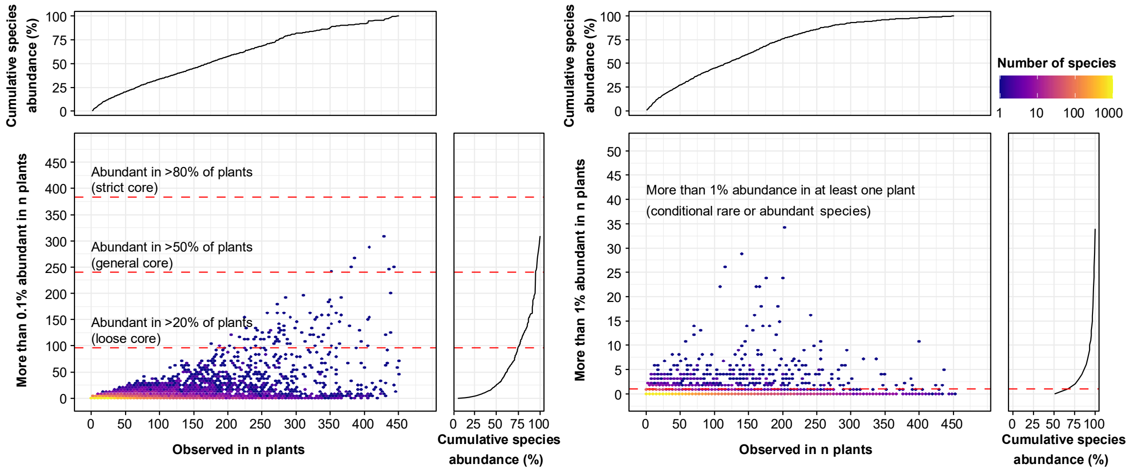

Supplementary Fig. 7: Identification of core and conditionally rare or abundant species based on V4 amplicon data. Identification of strict, general, and loose core species based on how often a given species was observed at a relative abundance above 0.1% in WWTPs. Identification of conditionally rare or abundant (CRAT) species based on whether a given species was observed at a relative abundance above 1% in at least one WWTP. The cumulative species abundance is based on all ASVs classified at the species-level. All core species have been removed before the identification of the CRAT species.

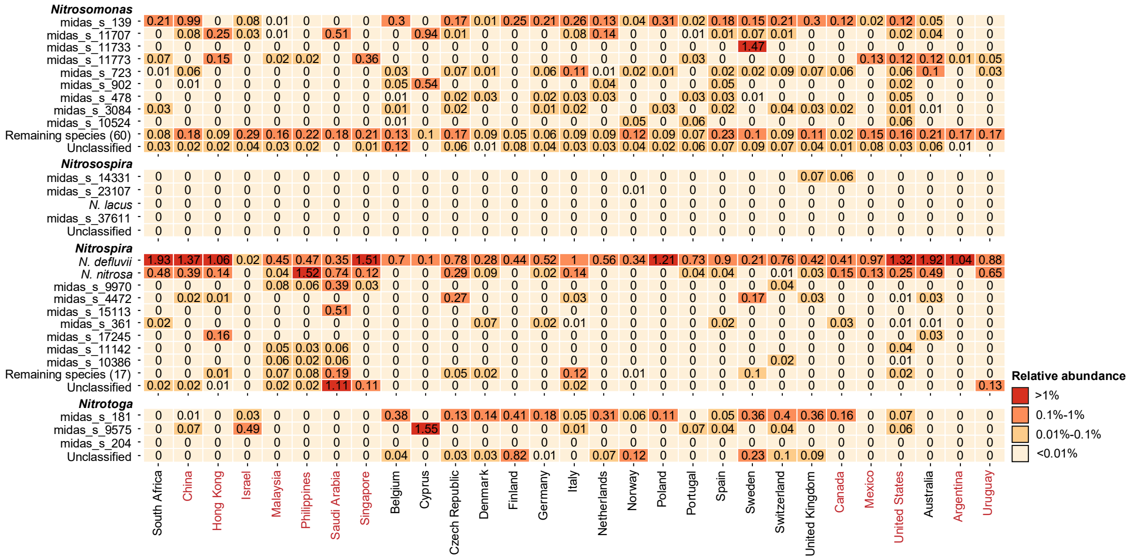

Supplementary Fig. 8: Global species-level diversity of nitrifiers. The percent relative abundance represents the mean abundance for each country taking into account only WWTPs with nitrification (C,N; C,N,DN, C,N,DN,P). Countries are grouped based on continent (shifting color).

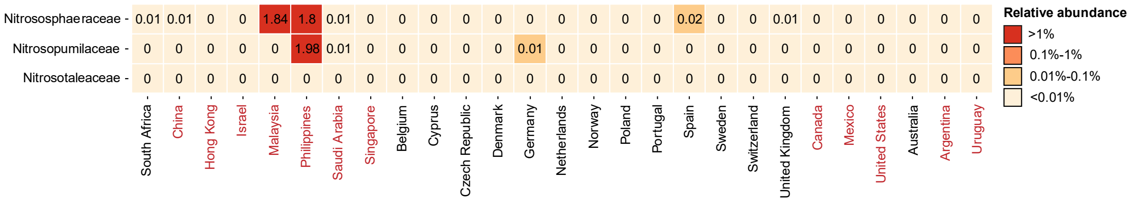

Supplementary Fig. 9: Global family-level diversity of ammonia oxidizing archaea (AOA). The percent relative abundance represents the mean abundance for each country taking into account only WWTPs with nitrification (C,N; C,N,DN, C,N,DN,P). V4 amplicon data classified with the SILVA SSURef 138 database was used for the analysis. Countries are grouped based on continent (shifting color).

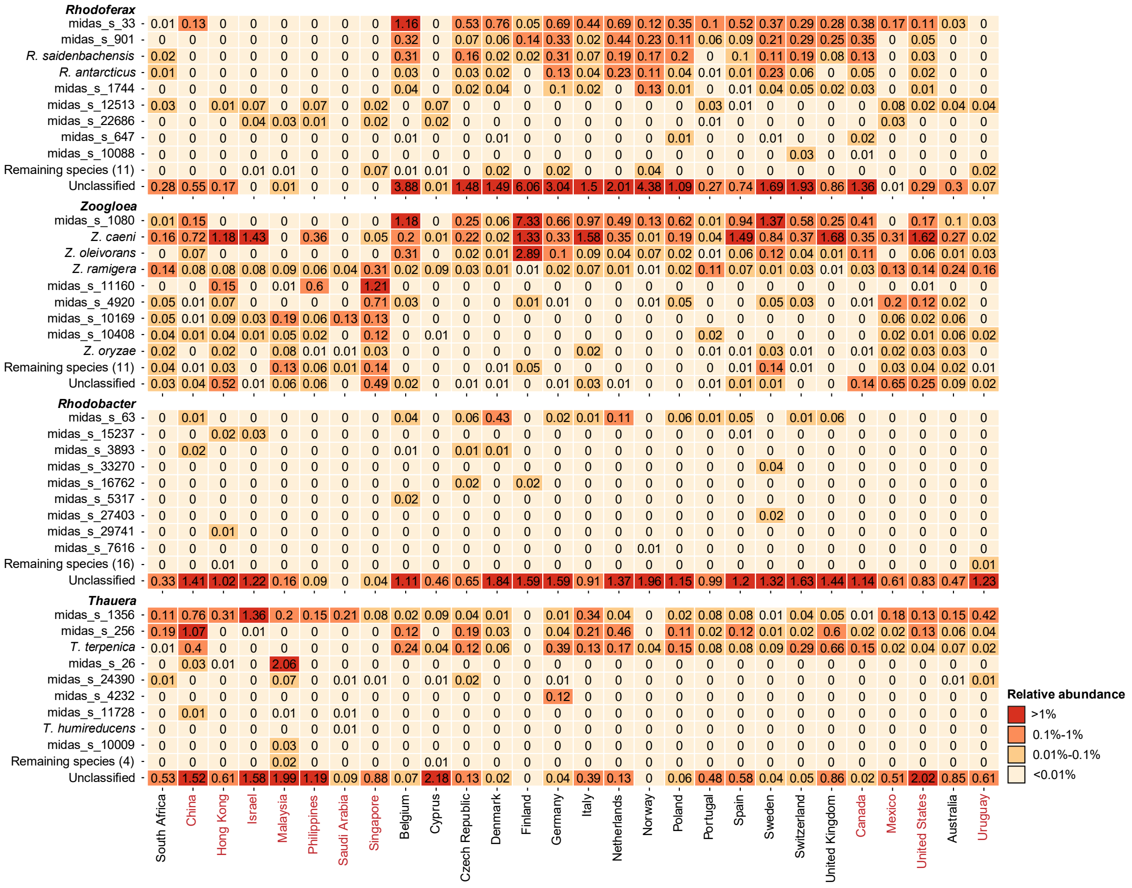

Supplementary Fig. 10: Global species-level diversity of top four denitrifiers. The percent relative abundance represents the mean abundance for each country taking into account only WWTPs with denitrification (C,N,DN, C,N,DN,P). Countries are grouped based on continent (shifting color).

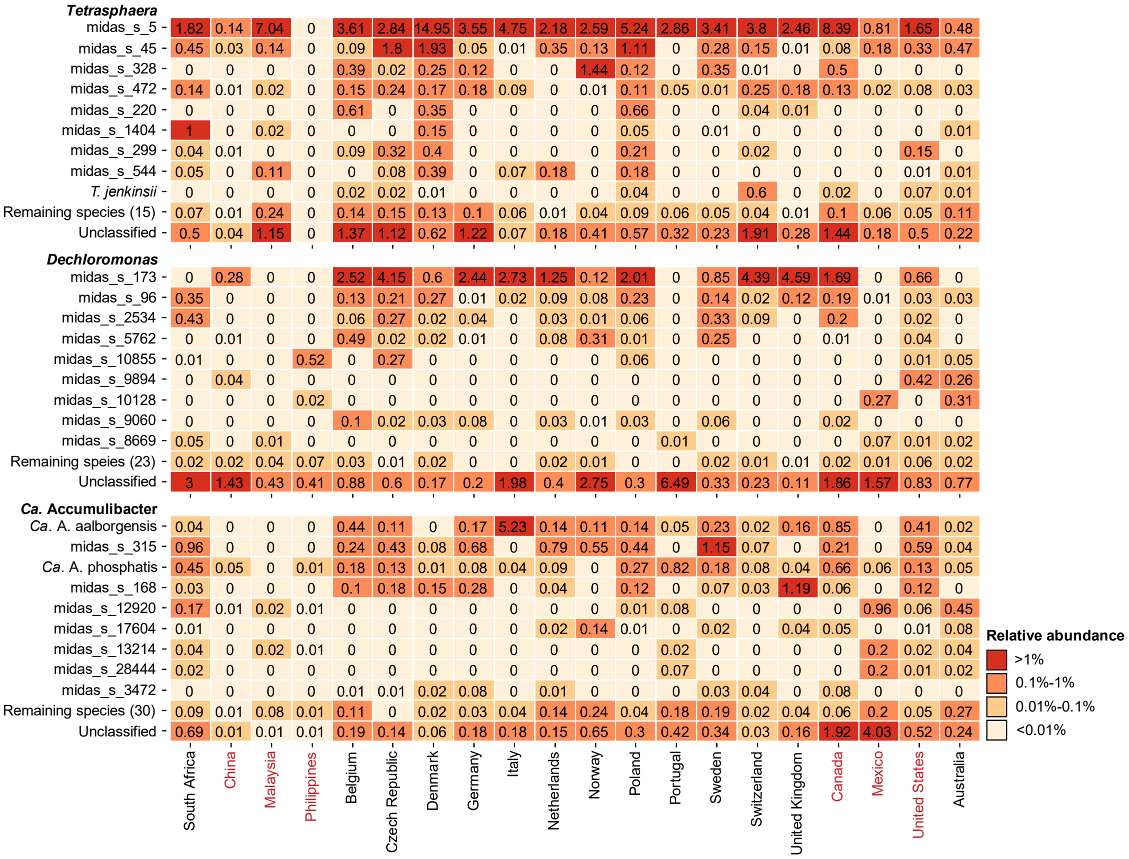

Supplementary Fig. 11: Global species-level diversity of PAOs. The percent relative abundance represents the mean abundance for each country taking into account only WWTPs with enhanced biological phosphorus removal (C,N,DN,P). Countries are grouped based on continent (shifting color).

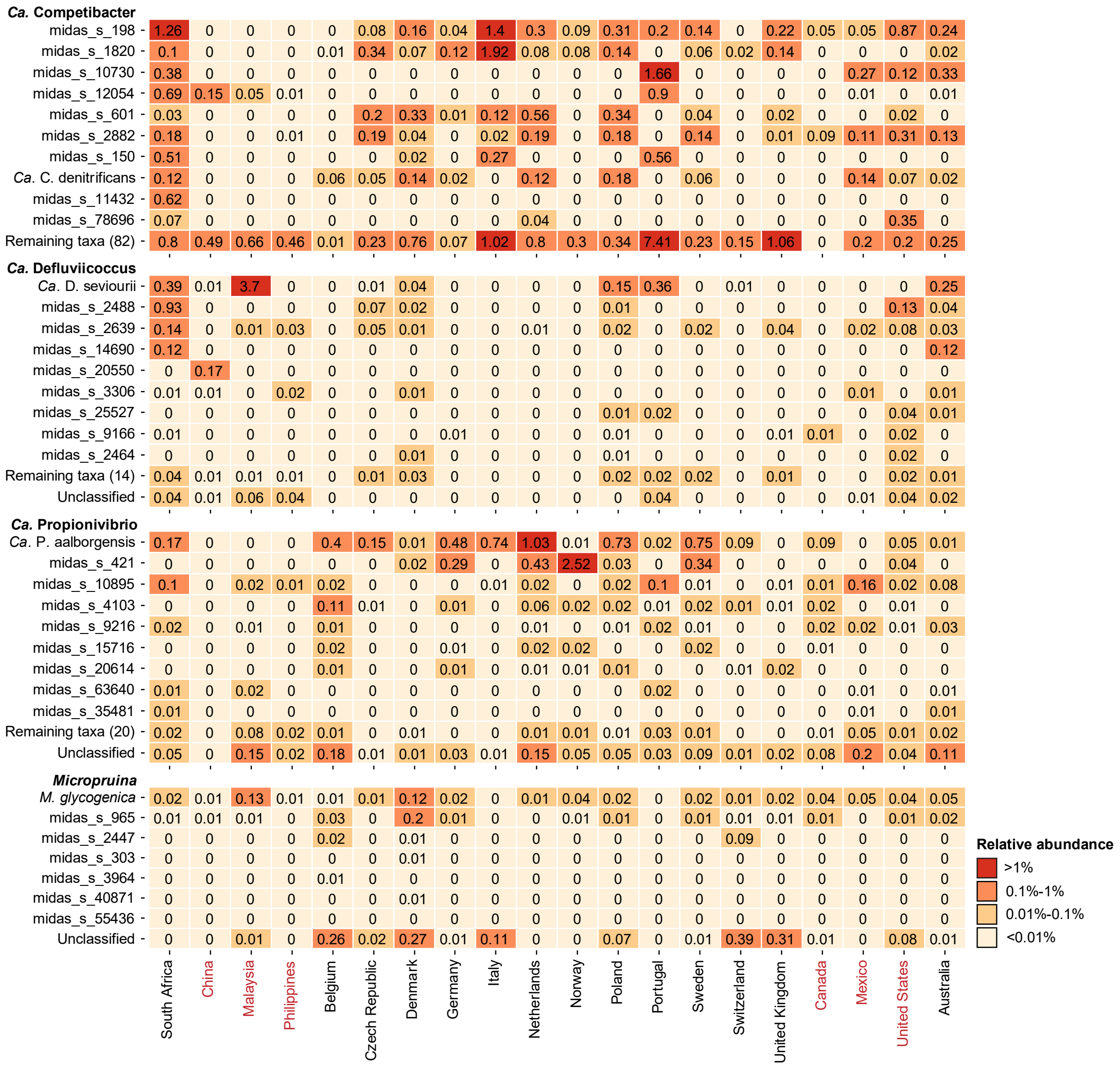

Supplementary Fig. 12: Global species-level diversity of GAOs. The percent relative abundance represents the mean abundance for each country taking into account only WWTPs with enhanced phosphorus removal (C,N,DN,P). Countries are grouped based on continent (shifting color).

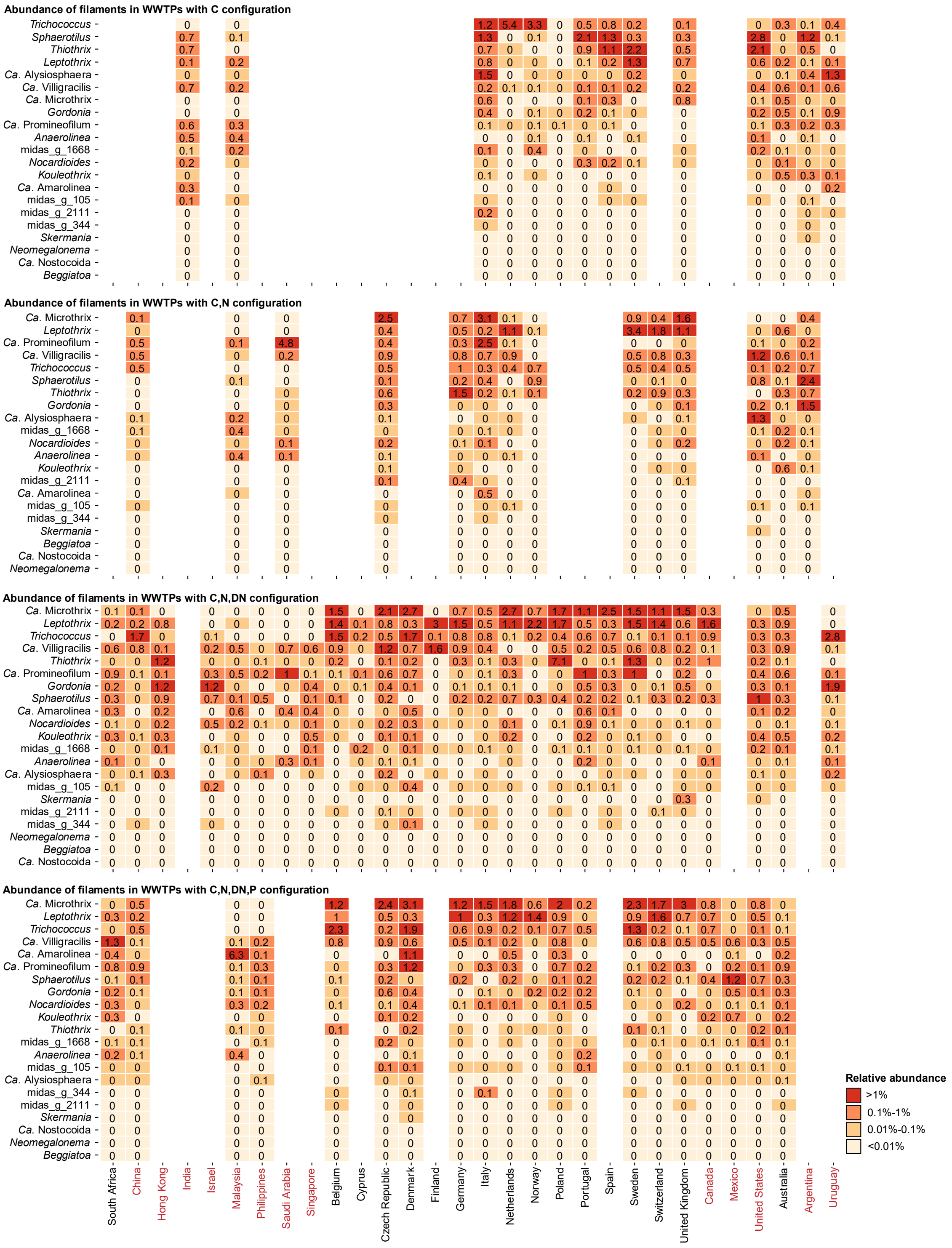

Supplementary Fig. 13: Global diversity of known filamentous organisms in different process types. The percent relative abundance represents the mean abundance for each country and each process type. Countries are grouped based on continent (shifting color).

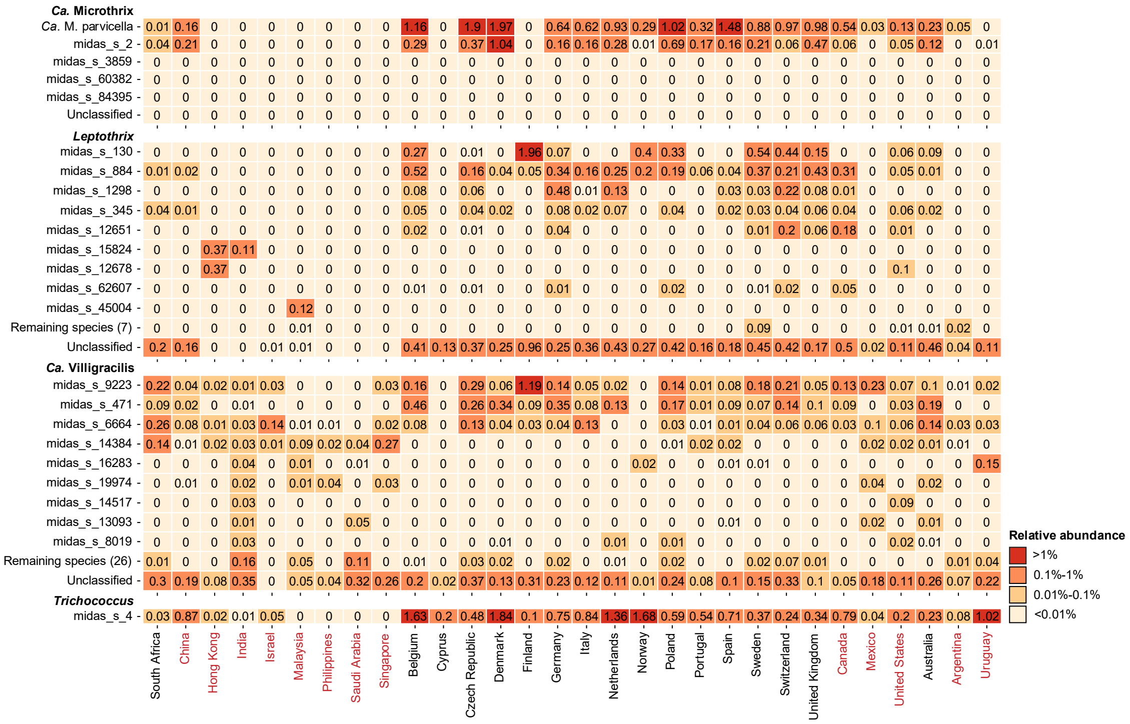

Supplementary Fig. 14: Global species-level diversity of top four filamentous genera. The percent relative abundance represents the mean abundance for each country. Countries are grouped based on continent (shifting color).

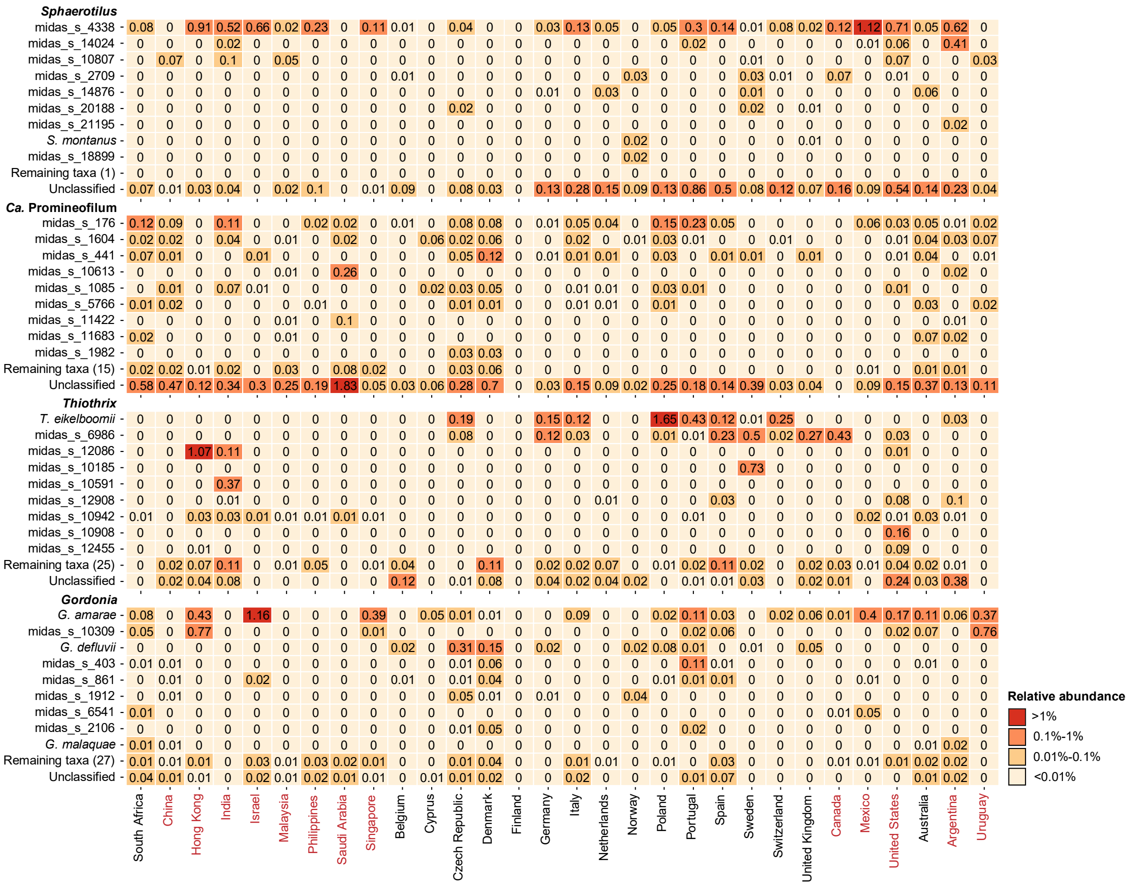

Supplementary Fig. 15: Global species-level diversity of filamentous genera (top 5-8). The percent relative abundance represents the mean abundance for each country. Countries are grouped based on continent (shifting color).

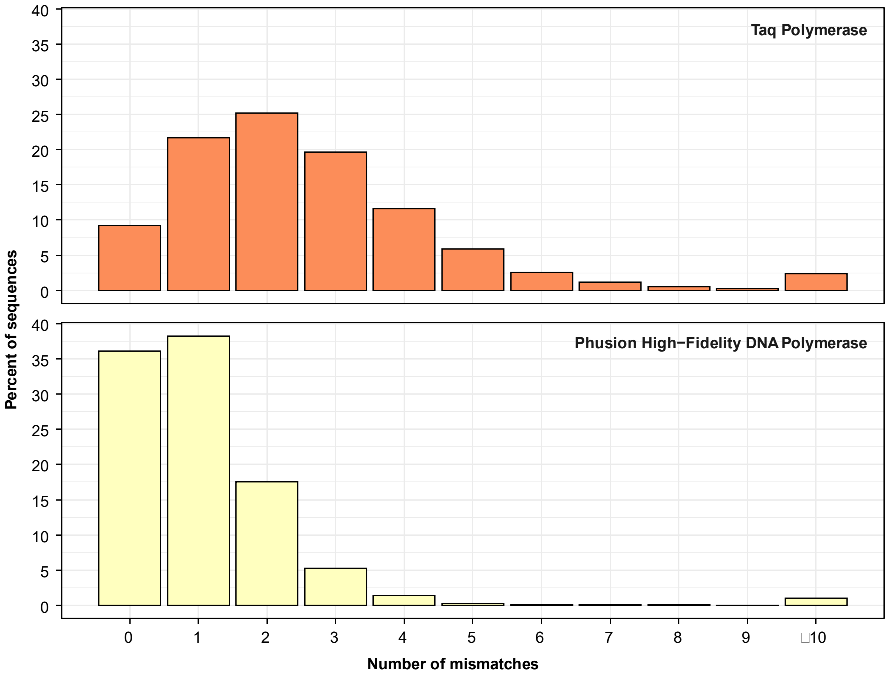

Supplementary Fig. 16: Replacement of the Taq polymerase with the Phusion HiFi DNA polymerase improves the raw synthetic long-read error rate. The libraries were created as described in the materials and methods except for the primary amplification with the Taq polymerase where the Phusion HF buffer (NEB) was replaced with QIAGEN PCR buffer and the 2 units of Phusion HF DNA polymerase (NEB) was replaced with 2.5 units of Taq polymerase (QIAGEN). The ZymoBIOMICS Microbial Community DNA Standard was used as a template and sequences were matched against the corrected reference sequences from Karst et al. 2021 [9].

Supplementary Tables

Supplementary Table 1: Oligonucleotides used for full-length 16S rRNA gene library preparation. Unique molecular tags and sample barcodes are marked with blue and red, respectively.

Name	Sequence	Protocol step
f16S_pcr1_fw1	CTCCACCCAGACTCATCCATNNNNNNNNNNNNNNNTGCCTCTTAGAGTTTGATCMTGGCTCAG	Barcoding and unique molecular
f16S_pcr1_fw2	CTCCACCCAGACTCATCCATNNNNNNNNNNNNNNNTCCTCTACAGAGTTTGATCMTGGCTCAG	identifier (UMI) tagging
f16S_pcr1_fw3	CTCCACCCAGACTCATCCATNNNNNNNNNNNNNNNTCATGAGCAGAGTTTGATCMTGGCTCAG	
f16S_pcr1_fw4	CTCCACCCAGACTCATCCATNNNNNNNNNNNNNNNCCTGAGATAGAGTTTGATCMTGGCTCAG	
f16S_pcr1_fw5	CTCCACCCAGACTCATCCATNNNNNNNNNNNNNNNTAGCGAGTAGAGTTTGATCMTGGCTCAG	
f16S_pcr1_fw6	CTCCACCCAGACTCATCCATNNNNNNNNNNNNNNNGTAGCTCCAGAGTTTGATCMTGGCTCAG	
f16S_pcr1_rv1	AGCGCGGCAAAGATGAAGATNNNNNNNNNNNNNNNTGAACCTTGACGGGCGGTGWGTRCA	
f16S_pcr1_rv2	AGCGCGGCAAAGATGAAGATNNNNNNNNNNNNNNNTGCTAAGTGACGGGCGGTGWGTRCA	
f16S_pcr2_fw	CTCCACCCAGACTCATCCAT	Library and clonal amplification
f16S_pcr2_rv	AGCGCGGCAAAGATGAAGAT	
f16S_readtag_fw	CAAGCAGAAGACGGCATACGAGATGTGACTGGAGTTCAGACGTGTGCTCTTCCGATCTCTCCACCCAGACTCATCCAT	Read-tag library preparation
f16S_readtag_rv	CAAGCAGAAGACGGCATACGAGATGTGACTGGAGTTCAGACGTGTGCTCTTCCGATCTAGCGCGGCAAAGATGAAGAT	
f16S_linktag_fw	CAAGCAGAAGACGGCATACGAGATCGGTCTCGGCATTCCTGCTGAACCGCTCTTCCGATCTCTGAGCCAKGATCAAACTCT	Linked-tag library preparation
f16S_linktag_rv	AATGATACGGCGACCACCGAGATCTACACTCTTTCCCTACACGACGCTCTTCCGATCTTGYACWCACCGCCCGTC	
f16S_read2_fw	GCTCTTCCGATCTCTCCACCCAGACTCATCCAT	Illumina HiSeq sequencing
f16S_read2_rv	GCTCTTCCGATCTAGCGCGGCAAAGATGAAGAT	

Supplementary References:

1. Seviour, R. J. & Nielsen, P. H. *Microbial Ecology of Activated Sludge*. (IWA Publishing, 2010).
2. Meerburg, F. A. *et al.* High-rate activated sludge communities have a distinctly different structure compared to low-rate sludge communities, and are less sensitive towards environmental and operational variables. *Water Res.* **100**, 137–145 (2016).
3. Wu, L. *et al.* Global diversity and biogeography of bacterial communities in wastewater treatment plants. *Nat. Microbiol.* **4**, 1183–1195 (2019).
4. Horner-Devine, M. C., Lage, M., Hughes, J. B. & Bohannan, B. J. M. A taxa–area relationship for bacteria. *Nature* **432**, 750–753 (2004).
5. Martiny, J. B. H., Eisen, J. A., Penn, K., Allison, S. D. & Horner-Devine, M. C. Drivers of bacterial β-diversity depend on spatial scale. *Proc. Natl. Acad. Sci.* **108**, 7850–7854 (2011).
6. Meyer, K. M. *et al.* Why do microbes exhibit weak biogeographic patterns? *ISME J.* **12**, 1404–1413 (2018).
7. Knights, D. *et al.* Rethinking "Enterotypes". *Cell Host Microbe* **16**, 433–437 (2014).
8. Layton, A. C. *et al.* Quantification of *Hyphomicrobium* populations in activated sludge from an industrial wastewater treatment system as determined by 16S rRNA analysis. *Appl. Environ. Microbiol.* **66**, 1167–1174 (2000).
9. Karst, S. M. *et al.* High-accuracy long-read amplicon sequences using unique molecular identifiers with Nanopore or PacBio sequencing. *Nat. Methods* **18**, 165–169 (2021).
