# Supplementary material for: MiDAS 4: A global catalogue of full-length 16S rRNA gene sequences and taxonomy for studies of bacterial communities in wastewater treatment plants
Source: Nat Commun. 2022 Apr 7;13:1908. doi: 10.1038/s41467-022-29438-7 (PMC8989995; doi:10.1038/s41467-022-29438-7)
Supplement: Supplementary file 2 — Reporting Summary [file 41467_2022_29438_MOESM2_ESM.pdf]

nature portfolio

Corresponding author(s): Per Halkjær Nielsen
Last updated by author(s): Mar 1, 2022

Reporting Summary

Nature Portfolio wishes to improve the reproducibility of the work that we publish. This form provides structure for consistency and transparency in reporting. For further information on Nature Portfolio policies, see our Editorial Policies and the Editorial Policy Checklist.

Statistics

For all statistical analyses, confirm that the following items are present in the figure legend, table legend, main text, or Methods section.

n/a	Confirmed	
☐	☒	The exact sample size (n) for each experimental group/condition, given as a discrete number and unit of measurement
☐	☒	A statement on whether measurements were taken from distinct samples or whether the same sample was measured repeatedly
☐	☒	The statistical test(s) used AND whether they are one- or two-sided<br>*Only common tests should be described solely by name; describe more complex techniques in the Methods section.*
☒	☐	A description of all covariates tested
☒	☐	A description of any assumptions or corrections, such as tests of normality and adjustment for multiple comparisons
☐	☒	A full description of the statistical parameters including central tendency (e.g. means) or other basic estimates (e.g. regression coefficient) AND variation (e.g. standard deviation) or associated estimates of uncertainty (e.g. confidence intervals)
☐	☒	For null hypothesis testing, the test statistic (e.g. F, t, r) with confidence intervals, effect sizes, degrees of freedom and P value noted<br>*Give P values as exact values whenever suitable.*
☒	☐	For Bayesian analysis, information on the choice of priors and Markov chain Monte Carlo settings
☒	☐	For hierarchical and complex designs, identification of the appropriate level for tests and full reporting of outcomes
☒	☐	Estimates of effect sizes (e.g. Cohen's d, Pearson's r), indicating how they were calculated

Our web collection on statistics for biologists contains articles on many of the points above.

Software and code

Policy information about availability of computer code

Data collection	External amplicon data fwas downloaded from NCBI Sequence Read Archive (SRA) using the SRA-Toolkit v.2.9.2.
Data analysis	All software used is described in the methods and includes:<br>Usearch v11.0.667,<br>R v4.0.5,<br>RStudio v1.4.1717,<br>tidyverse v1.3.1,<br>vegan v2.5,<br>maps v.3.3.0,<br>Ampvis2 v2.7.9,<br>CLC genomics workbench v20.0,<br>cutadapt v2.8,<br>RAxML v8.2.12,<br>ARB v6.0.6, and<br>SINA v.1.6<br><br>R-markdown scripts used for data analyses and figures are available at GitHub:<br>https://github.com/msdueholm/Publications/tree/master/Dueholm2022a

For manuscripts utilizing custom algorithms or software that are central to the research but not yet described in published literature, software must be made available to editors and reviewers. We strongly encourage code deposition in a community repository (e.g. GitHub). See the Nature Portfolio guidelines for submitting code & software for further information.

Data

Policy information about availability of data

All manuscripts must include a data availability statement. This statement should provide the following information, where applicable:

- Accession codes, unique identifiers, or web links for publicly available datasets
- A description of any restrictions on data availability
- For clinical datasets or third party data, please ensure that the statement adheres to our policy

Raw and assembled sequencing data is available at the NCBI SRA database with BioProject ID: PRJNA728873.
The MiDAS 4 reference database in SINTAX and QIIME format, is available at https://www.midasfieldguide.org/guide/downloads.
Raw data files for the RMarkdown scripts are available at figshare: https://doi.org/10.6084/m9.figshare.16566408.v1.

Field-specific reporting

Please select the one below that is the best fit for your research. If you are not sure, read the appropriate sections before making your selection.

☐ Life sciences ☐ Behavioural & social sciences ☒ Ecological, evolutionary & environmental sciences

For a reference copy of the document with all sections, see nature.com/documents/nr-reporting-summary-flat.pdf

Ecological, evolutionary & environmental sciences study design

All studies must disclose on these points even when the disclosure is negative.

Study description	The purpose of this study was to create a comprehensiv full-length 16S rRNA gene reference database and taxonomy for bacteria in wastewater treatment plants (WWTPs) across the world (MiDAS 4), and use this database in combination with short-read 16S rRNA gene amplicon data (V1-V3 and V4) to study how geographical and environmental factors shape the global microbiota in WWTPs.
Research sample	The samples used in this study are activated sludge or biomass from biofilters from WWTPs across the world. The samples contain the microbial communities that assimilate or transforms nutrients and polutants in the wastewater, thereby leading to a cleaned effluent. Due to the large number and diversity of WWTPs sampled, we believe that we have obtained reprentatives for almost all important bacteria in WWTPs globally.
Sampling strategy	To facilitate sampling, we established the MiDAS global consortium, which consists of 39 wastewater treatment experts in 31 countries. Members of the consortium acted as national sampling coordinators and were in direct contact with the WWTPs.<br><br>The WWTPs were selected based on the availablity of national sampling coordinators and their access to WWTPs. A total of 740 WWTPs in 425 cities, 31 countries on six continents was sampled. The majority of the WWTPs were configured with the activated sludge process, as this is the most common biological treatment process for wastewater treatment. However, WWTPs based on biofilters, moving bed bioreactors (MBBR), membrane bioreactors (MBR), and granular sludge were also included to cover the microbial diversity in other types of WWTPs. The samples were preserved in RNAlater during transportation and storage to minimize changes in the microbial composition.
Data collection	Samples were collected from the WWTPs by trained plant technicians according to guidelines provided by the authors. Samples were immediately shipped on ice to the national sampling coordinators who stabilised the samples with RNAlater and shipped them to the Center for Microbial Communities where they were processed by the authors.<br><br>The national coordinates also collected geographical and environmental metadata for the samples in excel templates prepared by the authors. Minimum information from all plants included continent, country, GPS coordinates, sampling date, temperature in the process tank, wastewater composition (municipal vs. industrial COD fraction), process type, and plant type.<br><br>Synthetic long-read 16S rRNA sequencing libraries were prepared by the authors and sequenced on a HiSeq2500 by Admera Health.<br><br>Short-read amplicon was produced by the authors and sequenced on a MiSeq in-house. Data was analysed at the Center for Microbial Communities by the authors.
Timing and spatial scale	All samples used for microbial community profiling were collected in 2018. They include biological replicates for each WWTP collected at the same time, but from different locations in the process tank. The WWTPs were not sampled at the same time of the year due to limited access to WWTPs and availablity of the national sampling coordinators. The study does not include temporal sampling of individual WWTPs because the main focus of the study is on the global diversity of microbes in WWTP and this is also observed across WWTPs. A few older samples from the MiDAS sample collection, representing specialised WWTPs, were also applied for full-length 16S rRNA gene sequencing to expand the coverage of the MiDAS 4 database.
Data exclusions	No data were excluded from the study. However, we mainly focus on activated sludge samples in the microbial community composition analyses, because the majority of the samples are from activated sludge and this is the most common WWTP configuration globally. This choice was made after we had obtained metadata for all samples. The limited number of samples from other plant configurations prevents us from performing meaningful statistical tests for these. However, these samples are important for the coverage of the MiDAS 4 database.

Reproducibility	The work is observational and discovery based. Experimental reproducibility was therefore not relevant to the study aims.
Randomization	Randomization is not relevant for the study, because samples are catagorized according the metadata provided by the WWTPs.
Blinding	Samples and metadata were independently collected for each WWTP by the national coordinators. Samples were assigned random identifiers, which were used during sample processing and data handling. Sample metadata was first reintroduced during the final data analyses.

Did the study involve field work? ☐ Yes ☒ No

Reporting for specific materials, systems and methods

We require information from authors about some types of materials, experimental systems and methods used in many studies. Here, indicate whether each material, system or method listed is relevant to your study. If you are not sure if a list item applies to your research, read the appropriate section before selecting a response.

Materials & experimental systems

n/a	Involved in the study
☒	☐ Antibodies
☒	☐ Eukaryotic cell lines
☒	☐ Palaeontology and archaeology
☒	☐ Animals and other organisms
☒	☐ Human research participants
☒	☐ Clinical data
☒	☐ Dual use research of concern

Methods

n/a	Involved in the study
☒	☐ ChIP-seq
☒	☐ Flow cytometry
☒	☐ MRI-based neuroimaging
