# Supplementary material for: MiDAS 4: A global catalogue of full-length 16S rRNA gene sequences and taxonomy for studies of bacterial communities in wastewater treatment plants
Source: Nat Commun. 2022 Apr 7;13:1908. doi: 10.1038/s41467-022-29438-7 (PMC8989995; doi:10.1038/s41467-022-29438-7)
Supplement: Supplementary file 4 — Description of Additional Supplementary Files [file 41467_2022_29438_MOESM4_ESM.pdf]

Title: Supplementary Data 1:
Description: Metadata for wastewater treatment plants.

Title: Supplementary Data 2:
Description: Comparison of relative genus abundance based on V1-V3 and V4 region 16S rRNA gene amplicon data.

Title: Supplementary Data 3:
Description: Scores from RDA analyses.

Title: Supplementary Data 4:
Description: Core and conditional rare and abundant taxa identified in the V1-V3 and V4 dataset.
